# Pulse Oximetry Overestimates Arterial Oxygen Saturation and Alters Fick-Derived Hemodynamics During Right Heart Catheterization

**DOI:** 10.1016/j.jscai.2026.105345

**Published:** 2026-04-24

**Authors:** Vagisha Sharma, Prima Wulandari, Asna Aafreen, Yang Hao, Edo Kaluski

**Affiliations:** a Department of Internal Medicine, Guthrie Robert Packer Hospital, Sayre, Pennsylvania; b Division of Cardiology, Guthrie Robert Packer Hospital, Sayre, Pennsylvania

Pulse oximetry (SpO_2_) is widely used as a surrogate for arterial oxygen saturation (SaO_2_); however, device and physiologic limitations can introduce systematic error, particularly in cardiopulmonary disease, low perfusion states, and across varying skin tones.^1^^,^^2^ In invasive hemodynamic assessment, this discrepancy is clinically relevant because SaO_2_ directly determines arterial oxygen content and therefore influences Fick-derived cardiac output (CO) and vascular resistance calculations.^3^ Despite this dependency, SpO_2_ is occasionally substituted for SaO_2_ during catheterization workflows when arterial sampling is unavailable or delayed. We sought to quantify the frequency and hemodynamic impact of SpO_2_–SaO_2_ discordance during routine right heart catheterization.

We performed a retrospective cohort analysis of 50 consecutive patients undergoing combined right and left heart catheterization with simultaneous pulse oximetry and direct SaO_2_ measurement between August 2023 and March 2024 at a single center. Patients demonstrating an SpO_2_–SaO_2_ difference ≥5 percentage points comprised the analytic cohort. This threshold was selected to capture clearly clinically meaningful discordance beyond expected device variability. Current regulatory performance standards for pulse oximeters generally target an accuracy root mean square of approximately ≤3% when comparing SpO_2_ with SaO_2_, meaning most readings fall within about ±2% to 3% of the true arterial saturation.^4^

Arterial and mixed venous oxygen saturations were obtained by co-oximetry using an Avoximeter 1000E (Werfen) from arterial and pulmonary artery samples, respectively, while continuous SpO_2_ monitoring was recorded using LNCS DCI sensors with Masimo technology (Masimo). Fick CO was calculated using directly measured SvO_2_ and hemoglobin, first with SaO_2_ (reference standard) and then recalculated substituting SpO_2_ for SaO_2_; pulmonary vascular resistance (PVR) and systemic vascular resistance (SVR) were derived accordingly. Continuous variables are summarized as mean ± SD with ranges.

Among 50 patients undergoing right heart catheterization with simultaneous SpO_2_ and SaO_2_ measurements, the mean SpO_2_–SaO_2_ difference across the full cohort was 2.4 ± 2.8%. Thirty-eight patients (76%) demonstrated close agreement between the 2 measurements (<5% difference), whereas 12 patients (24%) showed ≥5% discordance. Notably, no patients demonstrated discordance between 3% and 5%.

The 12 patients with ≥5% discordance comprised the analytic cohort. In this subgroup, SpO_2_ exceeded SaO_2_ by 9.3 ± 2.6% (range 5% to 13%), with a mean SpO_2_/SaO_2_ ratio of 1.11 (median 1.11; range 1.06-1.15), reflecting systematic overestimation of arterial oxygen saturation by pulse oximetry.

The mean age of the analytic cohort was 66.1 ± 12.7 years, and 5 patients (42%) were male. The cohort reflected typical referral physiology: chronic obstructive pulmonary disease in 9 patients (75%), obstructive sleep apnea in 7 (58%), and heart failure with preserved ejection fraction or heart failure with reduced ejection fraction in 5 (42%). The population was predominantly non-Hispanic White (11/12), with 1 non-Hispanic Black patient (Supplemental Table S1).

Substituting SpO_2_ for SaO_2_ resulted in a 30% mean underestimation of Fick CO (range 16%-44%), with parallel inflation of resistance estimates (Supplemental Table S2, Figure 1). Mean calculated SVR increased by 46% and PVR by 44% when SpO_2_ was substituted for SaO_2_.

**Figure 1:**
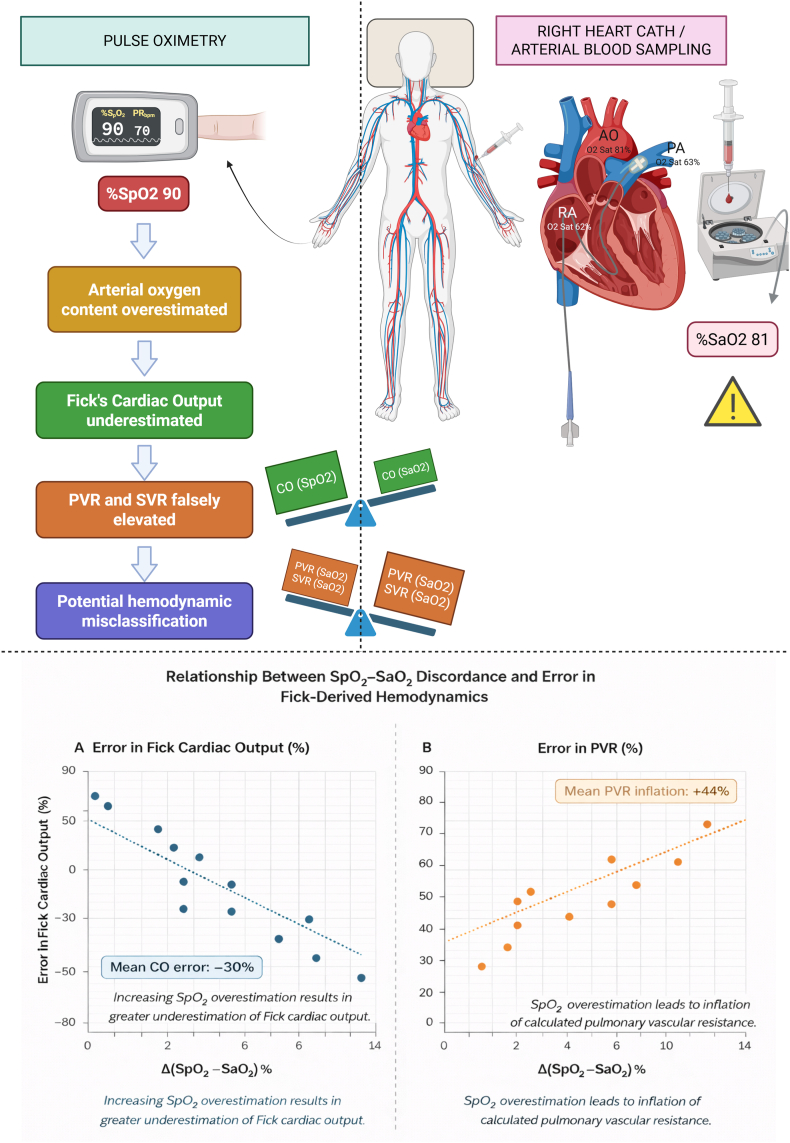
**Impact of Sp****O****_2_–Sa****O****_2_ discordance on Fick-derived hemodynamics.**The top panel illustrates how pulse oximetry overestimation of arterial oxygen saturation (SpO_2_) relative to directly measured arterial saturation (SaO_2_) leads to overestimation of arterial oxygen content, resulting in underestimation of Fick cardiac output and inflation of pulmonary and systemic vascular resistance. The bottom panels show the relationship between increasing SpO_2_–SaO_2_ discordance and error in derived hemodynamics. Panel A demonstrates progressive underestimation of Fick cardiac output (mean error −30%), whereas panel B shows increasing calculated pulmonary vascular resistance (mean inflation +44%). This figure was created with BioRender.com. AO, aorta; CO, cardiac output; PA, pulmonary artery; PVR, pulmonary vascular resistance; RA, right atrium; SVR, systemic vascular resistance.

This distortion is physiologically predictable and clinically meaningful. Pulse oximetry estimates saturation using 2-wavelength spectrophotometry from pulsatile arterial flow.^1^ Under conditions of reduced perfusion or altered light absorptio—such as increased skin melanin, motion artifact, or dyshemoglobinemia—devices may overestimate oxyhemoglobin proportion.^1^^,^^2^ Accordingly, recent US Food and Drug Administration draft guidance on pulse oximeter performance emphasizes diversity-inclusive accuracy testing, highlighting the importance of equitable device performance across patient populations.^5^

When SpO_2_ substitutes for SaO_2_ in the Fick equation, arterial oxygen content is overestimated, artifactually depressing calculated CO. Clinically, this bias may alter hemodynamic classification. In several patients, artifactually depressed Fick CO could simulate a low-output state and lead to unnecessary escalation of therapy. Similarly, substituting SpO₂ for SaO₂ increased calculated PVR beyond the 3 Wood unit threshold, potentially suggesting precapillary pulmonary hypertension.

Discrepancies appeared greatest among patients with overlapping cardiopulmonary disease, consistent with prior observations that ventilation–perfusion mismatch and impaired peripheral perfusion degrade oximeter fidelity.^2^ However, the limited sample size precluded formal statistical testing of this association.

In contemporary practice, isolated right heart catheterization is often performed without routine arterial sampling. At our institution, arterial blood gas sampling is routinely obtained when Fick-derived hemodynamic calculations are performed. Our findings suggest that reliance on pulse oximetry alone in such settings may introduce unrecognized error in calculated CO and vascular resistance, particularly when values lie near diagnostic thresholds.

Although prior studies have described discrepancies between SpO_2_ and SaO_2_ measurements, their downstream impact on invasive hemodynamic calculations during cardiac catheterization has been incompletely characterized.^2^^,^^4^ During right heart catheterization, arterial oxygen saturation should be obtained directly rather than inferred from pulse oximetry, particularly when Fick-derived CO, PVR, or SVR inform diagnostic or therapeutic decisions. Importantly, the implications extend beyond the catheterization laboratory. Given the widespread use of pulse oximetry to guide oxygen therapy and screening in settings such as overnight oximetry, overestimation may delay recognition of low oxygen levels, misclassify patients who require oxygen, and result in inappropriate or inadequate treatment. Our findings demonstrate how SpO_2_–SaO_2_ discordance can propagate into invasive hemodynamic calculations and clinical interpretation, underscoring a clinically important limitation with implications across multiple areas of patient care.

Limitations include the small sample size, single-center design, and selection of cases with larger saturation gaps; however, the consistent direction and magnitude of bias support a clinically meaningful phenomenon warranting validation in larger cohorts.

## CRediT authorship contribution statement

**Vagisha Sharma:** Conceptualization, Data curation, Formal analysis, Investigation, Methodology, Writing – original draft, Writing – review & editing. **Prima Wulandari:** Conceptualization, Data curation, Formal analysis, Writing – review & editing. **Asna Aafreen:** Conceptualization, Data curation, Formal analysis, Supervision, Validation. **Yang Hao:** Conceptualization, Data curation, Supervision, Validation, Visualization, Writing – review & editing. **Edo Kaluski:** Conceptualization, Data curation, Formal analysis, Investigation, Methodology, Project administration, Resources, Supervision, Validation, Writing – review & editing.

## Declaration of competing interest

The authors declared no potential conflicts of interest with respect to the research, authorship, and/or publication of this article.

## Footnotes

To access the supplementary material accompanying this article, visit the online version of the Journal of the Society for Cardiovascular Angiography & Interventions at 10.1016/j.jscai.2026.105345.
