# Supplementary material for: Pulse Oximetry Overestimates Arterial Oxygen Saturation and Alters Fick-Derived Hemodynamics During Right Heart Catheterization
Source: J Soc Cardiovasc Angiogr Interv. 2026 Apr 24;5(6):105345. doi: 10.1016/j.jscai.2026.105345 (PMC13404051; doi:10.1016/j.jscai.2026.105345)
Supplement: Supplementary Tables S1 [file mmc1.docx]

**Supplemental Table S1. Baseline Demographic and Clinical Characteristics of Patients With ≥5% SpO₂–SaO₂ Discordance Undergoing Right Heart Catheterization**

| Case | Age (y) | Sex | Race/Ethnicity | Clinical Indication | BMI (kg/m2) | Hgb (g/dL) | EF (%) | COPD | OSA | Nicotine smoking history | Major Comorbidity |
| --- | --- | --- | --- | --- | --- | --- | --- | --- | --- | --- | --- |
| 1 | 71 | F | Non-Hispanic, white | Mod–severe AS, positive ischemic stress test | 30 | 14.9 | 55-60 | Y | N | 50 PY, current | Aortic Stenosis with Regurgitation |
| 2 | 49 | F | Non-Hispanic, white | PFO closure + fibroelastoma | 31 | 9.5 | 60-65 | N | N | 25 PY, former | Stroke |
| 3 | 59 | F | Non-Hispanic, white | Severe PAD + exertional dyspnea | 27.3 | 11.8 | 65 | Y | N | 46 PY, current | Peripheral vascular disease |
| 4 | 61 | M | Non-Hispanic, white | Unstable angina + mod–severe AS | 35 | 14.0 | 60-65 | N | Y | 32 PY, former | Valvular Heart disease |
| 5 | 63 | F | Non-Hispanic, white | New HFrEF (EF 35%) | 18.8 | 15.0 | 35 | Y | Y | 23 PY, current | HFrEF |
| 6 | 65 | M | Non-Hispanic, white | Exertional dyspnea, CAD risk | 28.5 | 15.7 | 60-65 | Y | N | 55 PY, current | Peripheral vascular disease |
| 7 | 68 | M | Non-Hispanic, white | Pulmonary hypertension (Group I) | 43.9 | 17.5 | 65–70 | Y | Y | 25 PY, current | HFpEF |
| 8 | 42 | M | Non-Hispanic, white | Progressive SOB, CAD RFs | 33 | 16.6 | 45 | N | Y | 24 PY, current | HFpEF |
| 9 | 77 | M | Non-Hispanic, white | Severe symptomatic AS | 26.1 | 11.8 | 55 | Y | N | Never | Peripheral vascular disease |
| 10 | 81 | M | Non-Hispanic, Black | Dyspnea | 28.7 | 9.8 | 60-65 | Y | Y | 66 PY, former | HFpEF |
| 11 | 87 | M | Non-Hispanic, white | CAD, AS, MS | 30.4 | 15.9 | >75 | Y | Y | 30 PY, former | Severe Triple vessel CAD |
| 12 | 70 | F | Non-Hispanic, white | HFpEF, Coronary Artery Calcifications | 44 | 13.0 | 50 | Y | Y | 100 PY, current | HFpEF |

**Supplemental Table S2. Case-Level Hemodynamic Measurements Comparing SaO₂- and SpO₂-Derived Fick Calculations**

| **Case** | **SpO₂ (%)** | **SaO₂ (%)** | **SvO₂ (%)** | **Δ(SpO₂–SaO₂)** | **CO (SaO₂)** | **CO (SpO₂)** | **ΔCO (%)** | **PVR (SaO₂)** | **PVR (SpO₂ calc)** | **SVR (SaO₂)** | **SVR (SpO₂ calc)** |
| --- | --- | --- | --- | --- | --- | --- | --- | --- | --- | --- | --- |
| 1 | 94 | 82 | 67 | 12 | 7.9 | 4.39 | 44 | 2.3 | 4.14 | 10.2 | 18.4 |
| 2 | 95 | 86 | 67 | 9 | 6.35 | 4.32 | 32 | 1.0 | 1.47 | 13.4 | 19.7 |
| 3 | 95 | 87 | 62 | 8 | 5.66 | 4.29 | 24 | 1.9 | 2.51 | 12.0 | 15.8 |
| 4 | 91 | 80 | 55 | 11 | 5.94 | 4.12 | 31 | 1.99 | 2.87 | 13.2 | 19.0 |
| 5 | 90 | 81 | 63 | 9 | 5.67 | 3.78 | 33 | 4.0 | 6.0 | 16.2 | 24.3 |
| 6 | 94 | 88 | 65 | 6 | 5.03 | 3.98 | 21 | 2.5 | 3.16 | 16.6 | 21.0 |
| 7 | 95 | 86 | 67 | 9 | 7.1 | 4.82 | 32 | 2.0 | 2.95 | 10.5 | 15.5 |
| 8 | 99 | 88 | 70 | 11 | 6.6 | 4.1 | 38 | 1.55 | 1.57 | 13.0 | 13.2 |
| 9 | 94 | 89 | 64 | 5 | 7.15 | 5.3 | 26 | 1.0 | 1.35 | 12.8 | 17.3 |
| 10 | 98 | 85 | 62 | 13 | 7.3 | 4.25 | 42 | 3.8 | 6.5 | 11.0 | 18.9 |
| 11 | 99 | 87 | 68 | 12 | 6.8 | 4.97 | 27 | 1.0 | 1.37 | 13.2 | 18.1 |
| 12 | 90 | 84 | 48 | 6 | 4.5 | 3.8 | 16 | 3.0 | 3.5 | 15.0 | 17.8 |

Abbreviations: AS = aortic stenosis; CAD = coronary artery disease; COPD = chronic obstructive pulmonary 2disease; EF = ejection fraction; HFrEF = heart failure with reduced ejection fraction; MAP = mean arterial pressure; MS = mitral stenosis; OSA = obstructive sleep apnea; PAD = peripheral arterial disease; PFO = patent foramen ovale; PVR = pulmonary vascular resistance; PY = Pack years; SVR = systemic vascular resistance; WU = Wood units.
